# Synthesis and Properties of Sucrose- and Lactose-Based Aromatic Ester Surfactants as Potential Drugs Permeability Enhancers

**DOI:** 10.3390/ph16020223

**Published:** 2023-02-01

**Authors:** Michele Verboni, Diego Romano Perinelli, Carol Yingshan Qiu, Mattia Tiboni, Annalisa Aluigi, Simone Lucarini, Jenny K. W. Lam, Andrea Duranti

**Affiliations:** 1 Department of Biomolecular Sciences, University of Urbino Carlo Bo, Piazza del Rinascimento 6, 61029 Urbino, PU, Italy; 2 School of Pharmacy, University of Camerino, Via Gentile III da Varano, 62032 Camerino, MC, Italy; 3 Department of Pharmacology and Pharmacy, LKS Faculty of Medicine, The University of Hong Kong, 21 Sassoon Road, Pokfulam, Hong Kong SAR, China; 4 Department of Pharmaceutics, UCL School of Pharmacy, University College London, 29-39 Brunswick Square, London WC1N 1AX, UK

**Keywords:** glycolipids, sucrose monoesters, lactose monoesters, sugar-based surfactants, biocompatibility studies, permeability enhancers

## Abstract

The delivery of therapeutics across biological membranes (e.g., mucosal barriers) by avoiding invasive routes (e.g., injection) remains a challenge in the pharmaceutical field. As such, there is the need to discover new compounds that act as drug permeability enhancers with a favorable toxicological profile. A valid alternative is represented by the class of sugar-based ester surfactants. In this study, sucrose and lactose alkyl aromatic and aromatic ester derivatives have been synthesized with the aim to characterize them in terms of their physicochemical properties, structure–property relationship, and cytotoxicity, and to test their ability as permeability enhancer agents across Calu-3 cells. All of the tested surfactants showed no remarkable cytotoxic effect on Calu-3 cells when applied both below and above their critical micelle concentration. Among the explored molecules, lactose *p*-biphenyl benzoate (URB1420) and sucrose *p*-phenyl benzoate (URB1481) cause a reversible ~30% decrease in transepithelial electrical resistance (TEER) with the respect to the basal value. The obtained result matches with the increased in vitro permeability coefficients (P_app_) calculated for FTIC-dextran across Calu-3 cells in the presence of 4 mM solutions of these surfactants. Overall, this study proposes sucrose- and lactose-based alkyl aromatic and aromatic ester surfactants as novel potential and safe permeation enhancers for pharmaceutical applications.

## 1. Introduction

Nowadays, many diseases are managed using therapies that mostly require injection-only administration. The option to move to a non-invasive delivery route is highly attractive because of the possibility to increase patient compliance, thereby allowing an easier procedure [1]. Drug delivery technologies that respond to this need are scarce, due to the very low permeation of drugs through mucosal surfaces, which usually represent a barrier to the passage of active compounds from the external environment into the systemic circulation [2,3]. Absorption enhancing agents are commonly utilized to increase the mucosal permeation of macromolecules; however, some of them show unacceptable toxicity profiles [4,5]. The finding of innovative absorption enhancers with a favorable toxicological profile that can effectively improve the mucosal absorption of drugs is therefore an urgent need, and their discovery continues among new chemical classes [6,7,8].

Sugar-based surfactants have attracted considerable attention from formulation scientists in the past few years due to their high biocompatibility and biodegradability [9]. They can be largely employed in different fields such as cosmetics, food, and pharmaceuticals [10,11]. Their broad applicability relies on the amphiphilic nature, endowed by the presence of a polar head (e.g., lactose, sucrose, and mannose) and a hydrophobic tail. Various modifications, both on the polar head portion (mainly changing the sugar type) and on the non-polar tail, have been explored and several molecules have been designed to obtain large classes of active amphiphilic surfactants with functional properties [12]. Among all of them, sugar-based fatty acid esters are the most common and easy to find on the market due to their many investigated applications. In particular, the emulsifying and permeability-enhancing ability as well as the antimicrobial and antibiofilm properties of lactose-based esters with saturated and unsaturated fatty acid have been described previously by our group [13,14,15,16,17,18,19]. Moreover, we synthesized a series of lactose-based monoesters bearing saturated C10, C12, C14, or C16 acyl chains and evaluated cytotoxicity and the ability to decrease transepithelial electrical resistance (TEER) on airway epithelium Calu-3 cells. Among all of the tested surfactants, 6**′**-lactose laurate was recognized as the most promising compound, as it induced a marked and reversible decrease in TEER (down to ~25% of the baseline value) on Calu-3 cells at a non-cytotoxic concentration [20]. Recently, we synthesized lactose- and other sugar-based esters with alkyl aromatic and aromatic tails and endowed with antimicrobial activities, as well as the inhibition of biofilm formation activities [21]. We were then interested in understanding whether this novel class of alkyl aromatic and aromatic derivatives of lactose and sucrose esters could potentially find applications as permeability enhancers across the mucosa in comparison to the lactose-based monoesters bearing saturated or unsaturated linear chains.

Therefore, in this study, alkyl aromatic and aromatic analogues of sucrose- and lactose-based surfactants were considered with the aim to characterize them and to evaluate their biocompatibility properties, such as those related to permeability enhancement across Calu-3 cells, as a model of the airway epithelium. Sucrose and lactose were selected for the polar head, meanwhile the hydrophobic tails were explored among three different substituents based on arylalkyl and aryl portions.

## 2. Results and Discussion

### 2.1. Chemistry

Aryl aromatic and aromatic lactose- and sucrose-based esters were designed, synthesized, and explored for the first time for the applications described herein. With regard to the procedures through which the surfactants were obtained, whereas those of lactose derivatives were previously described [21], those of sucrose-based derivatives were carried out by means of a modified Mitsunobu reaction [22,23]. With respect to the latter method, a lower quantity of aromatic acid (1.5 eq.) was used to obtain the monoesterification in position 6 with a reduction in the formation of undesired products.

For all sugar-based surfactants, physicochemical properties such as hydrophilic-lipophilic balance (HLB) and octanol–water portion coefficient (clogP) were also calculated (Table 1). All of the compounds could be classified as hydrophilic surfactants (HLB > 10) and could act as oil-in-water emulsifiers.

### 2.2. Thermogravimetric Analysis (TGA) and Differential Thermal Analysis/Scanning (DTA/DSC) Measurements

**Figure 1:**
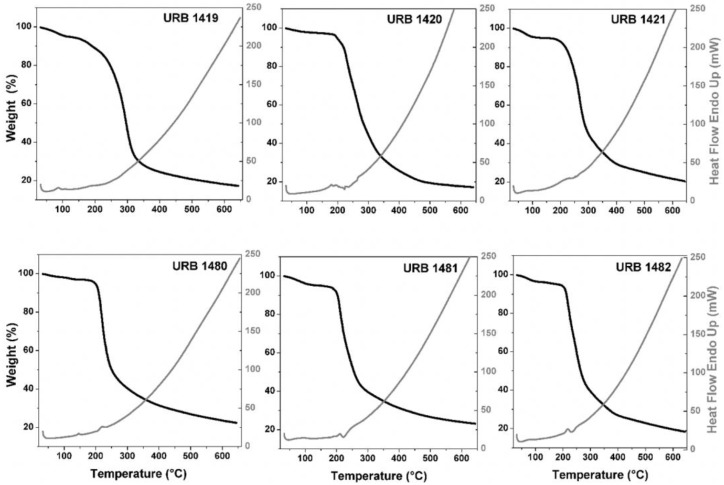
TGA and DTA of lactose-based surfactants (URB1419–URB1421) and sucrose-based surfactants (URB1480–URB1482). The black line in all of the plots refers to the “weight” signal, while the gray line in all of the plots refers to the “heat flow” signal.

All TGA traces showed two thermal events related to the weight loss of surfactants (Figure 1). The first one (≤5% of the initial mass), occurring in the temperature range of 50–125 °C, was related to water desorption of the absorbed or trapped water. The other one (~65–70% of the initial mass), occurring in the temperature range of 200–400 °C, was associated with the thermal degradation of surfactants. DTA/DSC profiles did not display any remarkable endothermic transition at temperatures lower than those at which degradation occurred, suggesting the possible amorphous nature or the simultaneous melting and degradation of the compounds. Overall, all alkyl aromatic analogues of sucrose- and lactose-based surfactants displayed similar thermal properties.

### 2.3. Critical Micelle Concentration (CMC) Measurements

**Figure 2:**
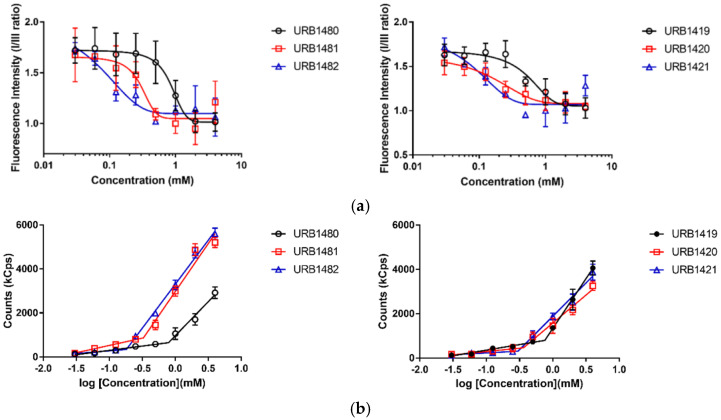
(**a**) Fluorescence intensity (peak I, III) vs. concentration plots for sucrose-based surfactants (URB1480-URB1482) and lactose-based surfactants (URB1419-URB1421). The values reported are the mean ± SD of three independent experiments. (**b**) Counts (kCps) vs. concentration plots from DLS measurements for sucrose-based surfactants (URB1480-URB1482) and lactose-based surfactants (URB1419-URB1421). The values reported are the mean ± SD of three independent experiments.

Figure 2a reports the variation of pyrene fluorescence emission (I and III peaks ratio) over concentration for all of the analyzed surfactants. Pyrene is a common fluorescence probe, widely employed to investigate the aggregation state of surfactant in aqueous solution, as its emission properties are strongly influenced by the polarity of the microenvironment. Specifically, a decrease in the ratio between I and III peaks is observed when the hydrophobicity increases in the surroundings of pyrene as a result of the aggregation of surfactants into micelles or supramolecular aggregates [26]. All plots show a sigmoidal decrease in the I and III pyrene peaks over concentration, denoting that all surfactants can self-assemble in H_2_O. As is common for surfactants, the concentration at which aggregation in aqueous solution occurs is strongly dependent on the hydrophilicity/hydrophobicity balance between the surfactant head and tail [27]. Specifically, changes in the hydrophobicity of the surfactant tail exert a marked effect on the self-assembling properties for a surfactant bearing the same polar head [28]. On the contrary, the variation in the sugar head has a minor effect [28]. As such, the sigmoidal profiles in Figure 2a are shifted toward a lower concentration from URB1480–URB1482 for sucrose-based surfactants and URB1419–URB1421 for lactose-based ones. This trend reflects the different hydrophobicity of the molecules related to the presence of a phenyl (URB1480 and URB1419) or a biphenyl group (URB1481, URB1482, URB1420, and URB1421) in the hydrophobic tail, a situation that affects CMC values (Table 2).

Indeed, the calculated CMC values were higher for the two surfactants bearing a phenyl group (URB1480 and URB1419) than for the others. For those surfactants with a biphenyl group, the elongation of the hydrophobic tail by the insertion of a methylene linker between the phenyl (hydrophobic chain) and sugar (polar head) moieties caused a further decrease in CMC. No marked differences in CMC values were observed between the sucrose-based and lactose-based series, underlining the less pronounced effect on CMC exerted by the sugar, as previously observed for other sugar-based surfactants [9].

The DLS from the counts analysis confirmed the self-assembling properties of the amphiphiles analyzed as evidenced by the fluorescence measurements. Figure 2b reports the variation in the scattering intensities to the detector (kCps) as a function of the surfactant concentration.

All the plots represented displayed an inflection point, corresponding to CMC, as a sudden increase in the measured counts of the solution occurred when the surfactants started to aggregate into the micelles. As unimers, instead, the hydrodynamic sizes of the surfactant molecules were too small and the scattering properties of the solutions were not markedly different from those of the medium [20,29].

### 2.4. Cytotoxicity—[3-(4,5-Dimethylthiazol-2-yl)-2,5-diphenyl tetrazolium bromide (MTT) Cell Viability and Lactate Dehydrogenase (LDH) Assays

**Figure 3:**
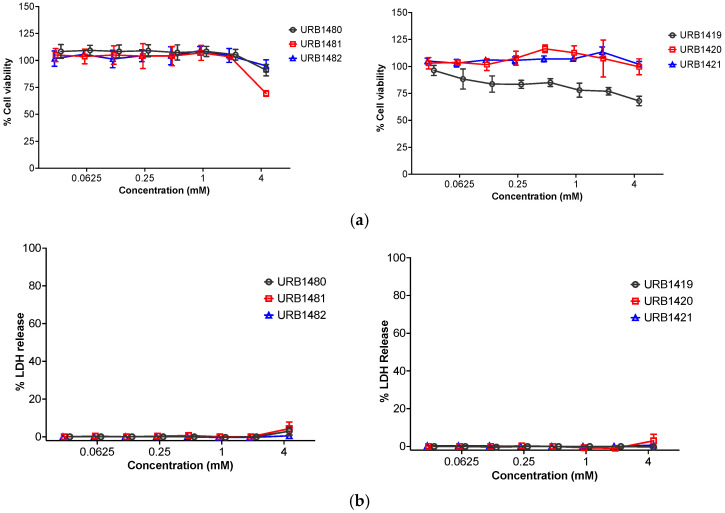
(**a**) MTT cell viability study of sucrose-based surfactants (URB1480-URB1482) and lactose-based surfactants (URB1419-URB1421) on Calu-3 cells. The values reported are the mean ± SD of three independent experiments. (**b**) LDH release study of sucrose-based surfactants (URB1480-URB1482) and lactose-based surfactants (URB1419-URB1421) on Calu-3 cells. The values reported are the mean ± SD of three independent experiments.

All the compounds were tested to evaluate their cytotoxicity profile on Calu-3 cells. The MTT colorimetric assay, based on the reduction of a yellow tetrazolium salt MTT to purple formazan crystals by metabolically active cells, was used to measure the cellular metabolic activity as an indicator of cell viability, proliferation, and cytotoxicity. Indeed, the viable cells contain NAD(P)H-dependent oxidoreductase enzymes, which reduce MTT to formazan [30]. The compounds did not show changes in the cell viability at all of the concentrations tested except for URB1481, which showed a reduction in viability to just above 70% at the highest tested concentration of 4.5 mM, and URB1419, which produced a decrease in viability of not lower than 70% (Figure 3a). Moreover, the LDH release assay, used to assess the level of plasma membrane damage in a cell population, was performed to evaluate the cytotoxicity of the sugar esters. LDH is in fact a stable enzyme found in all cell types and is rapidly released into the cell culture medium following damage to the plasma membrane that occurs after cell damage or death [31]. The results confirmed the high cell compatibility of the studied compounds at the tested concentrations (Figure 3b).

### 2.5. TEER Study

**Figure 4:**
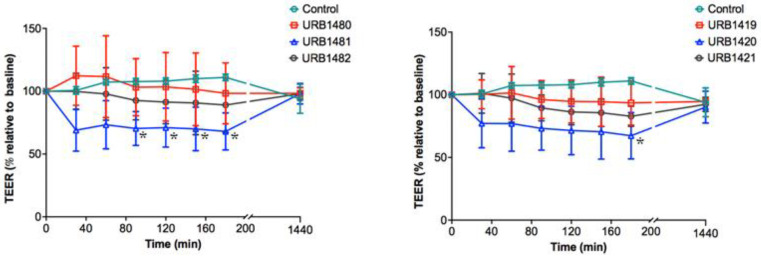
TEER study of sucrose-based surfactants (URB1480-URB1482) and lactose-based surfactants (URB1419-URB1421) at a concentration of 4 mM on Calu-3 monolayers. The values reported are the mean ± SD of three independent experiments. * *p* < 0.05 vs. control (Student’s *t*-test).

TEER studies were performed in Calu-3 cells (Figure 4) to preliminarily evaluate the potential use of the synthesized sugar esters as absorption enhancers. Data showed that URB1420 and URB1481 were the most effective surfactants for decreasing TEER, while URB1419, URB1421, and URB1482 showed a moderate action. URB1480 only poorly affected TEER, suggesting a possible reduced efficacy in tight junction opening or other membrane perturbation mechanisms [32].

The ability of URB1420 and URB1481 to lower TEER more than other synthesized surfactants suggests that the presence of the *p*-phenyl benzoate moiety as the hydrophobic tail seems to play a role through the permeation enhancing effect. On the contrary, if the *p*-biphenyl portion is linked to the sugar not directly but through a methylene spacer, as well as when a benzyl group linked to the ester group is present, the molecule decreases its ability to lower TEER. It is plausible to suppose that the presence of a flexible spacer could induce a conformation change in the biphenyl portion, such as to compromise the goodness of the action. The very small difference in the calculated values of HLB and clogP (Table 1) between the phenyl acetate and *p*-biphenyl acetate sugars does not allow us to think that these parameters can influence the obtained results. With regard to the toxicity profile, URB1420 demonstrated a good balance between safety and efficacy. Interestingly, TEER reversed to the initial value after 24 h with all the tested surfactants at a concentration of 4 mM, suggesting a transient effect on the Calu-3 monolayers and no cellular damage. The results obtained from the MTT assay support the TEER measurements, which should be interpreted carefully to ensure that the changes in TEER are not due to the permeant damage of the cellular membrane integrity. In fact, a transient modulation of the tight junction opening commonly translates into a reversible effect on TEER, while a permanent perturbation of the membrane integrity is evidenced by a non-reversible effect on TEER. However, it should also be noted that a reversible effect on TEER could be a consequence of a mechanism other than the tight junction opening [33,34]. By comparing the effect on TEER of the lactose-based monoesters versus that of the saturated linear derivatives, it should be noted that alkyl aromatic and aromatic lactose monoesters are less effective when used at non-cytotoxic concentrations. Indeed, URB1420 at the concentration of 4 mM induced a decrease in TEER on Calu-3 cells comparable to that of lactose caprate (C10) and lactose palmitate (C12) monoesters, when used at concentrations of 1 mM and 0.054 mM, respectively [20].

As several studies reported in the literature have demonstrated that a TEER decrease is correlated with an increase in FITC dextran flux across Calu-3 monolayers [35,36,37], a macromolecule permeability assay was then performed using FITC-dextran in order to collect some preliminary evidence whether the URB1420 and URB1481 could serve as permeation enhancers.

### 2.6. Permeability Study

**Figure 5:**
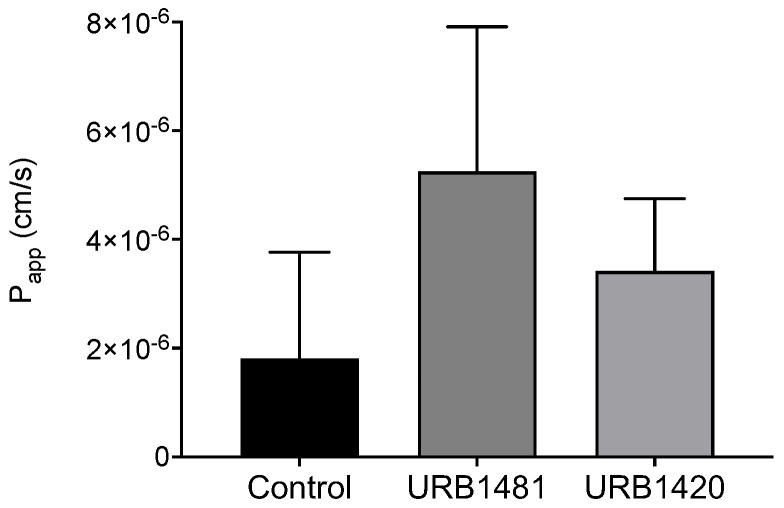
The apparent permeability coefficient of FITC-Dextran in the presence of URB1481 and URB1420 at a concentration of 4 mM across Calu-3 monolayers. The values reported are the mean ± SD of three independent experiments. * *p* < 0.05 vs. control (Student’s *t*-test).

The apparent permeability coefficient (Papp) values of Fluorescin isothiocyanate (FITC)-dextran in the absence (control) and in the presence of URB1481 and URB1420 at a concentration of 4 mM was calculated across Calu-3 cell layers (Figure 5). In accordance with TEER measurements, both surfactants resulted in an increase in FITC-dextran permeation. Comparing the two surfactants, URB1481 showed a more prominent permeation enhancing effect, while URB1420 demonstrated a moderate permeation that enhanced the effect but with a better safety profile. Further investigation into the permeation enhancer potential of both surfactants on other molecules and mucosal epithelial cells is warranted.

## 3. Materials and Methods

### 3.1. Materials

Sucrose and diisopropyl azodicarboxylate (DIAD) were purchased from Fluorochem (Hadfield, UK). Triphenylphosphine (PPh_3_) and oxalyl chloride [(CO)_2_Cl_2_] were purchased from Alpha Aesar (Ward Hill, MA, USA). Lactose monohydrate, *p*-toluene sulfonic acid, 2,2-dimethoxypropane, tetrafluoro boric acid diethyl ether complex [HBF_4_^.^Et_2_O], phenylacetic acid, *p*-biphenyl acetic acid, *p*-phenyl benzoic acid, acetone [CH_3_C(O)CH_3_], dimethylformamide (DMF), dimethyl sulfoxide (DMSO), methanol (CH_3_OH), and methylene chloride (CH_2_Cl_2_) were purchased from Sigma-Aldrich (Milan, Italy).

### 3.2. Synthesis of Sugar-Based Surfactants

**Figure 6:**
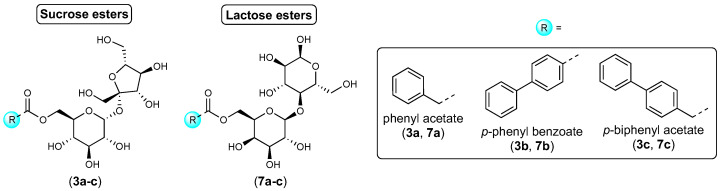
Chemical structures of sugar-based aromatic ester surfactants.

The structures of sugar-based aromatic esters (Figure 6) were unambiguously assessed by MS, ^1^H NMR, and ^13^C NMR. The ESI-MS spectra were recorded with a Waters Micromass^®^ ZQ™ (Waters Corporation, Milford, MA, USA) spectrometer in negative or positive mode using a nebulizing nitrogen gas at 400 L/min and a temperature of 250 °C, cone flow of 40 mL/min, capillary of 3.5 kV, and cone voltage of 60 V; only the molecular ions [M − H]^−^, [M + NH_4_]^+^, [M + Na]^+^, and [M + HCOO]^−^ were given. ^1^H NMR and ^13^C NMR spectra were recorded on a Bruker AC 400 or 101 (Bruker, Billerica, MA, USA) spectrometer, respectively, and were evaluated using the TopSpin 2.1 software package; chemical shifts were determined using the central peak of the solvent. Column chromatography purifications were performed under “flash” conditions using Merck 230–400 mesh silica gel (Darmstadt, Germany). Thin layer chromatography was carried out on Merck silica gel 60 F254 plates, which were visualized by exposure to ultraviolet light and to an aqueous solution of ceric ammonium molybdate.

#### 3.2.1. General Procedure for the Synthesis of Sucrose Aryl Aromatic and Aromatic Ester Surfactants (**3a–c**, URB1480–1482) (Scheme 1) [23]

Sucrose (**2**, 0.50 mmol) was dissolved in dry DMF (3.95 mL) at 70 °C under stirring and a nitrogen atmosphere. The mixture was cooled at room temperature, then PPh_3_ (0.354 g, 1.35 mmol), the appropriate carboxylic acid **1a**–**c** (0.75 mmol), and dry DMF (1.05 mL) were added. After complete dissolution, the mixture was cooled to 0 °C, and DIAD was added (0.256 mL, 1.35 mmol) dropwise, stirred for 24 h at room temperature, and concentrated. Purification of the residue by column chromatography [CH_2_Cl_2_/CH_3_C(O)CH_3_/CH_3_OH/H_2_O 78:10:10:1.5] resulted in **3a**–**c** as solids.

**Scheme 1:**
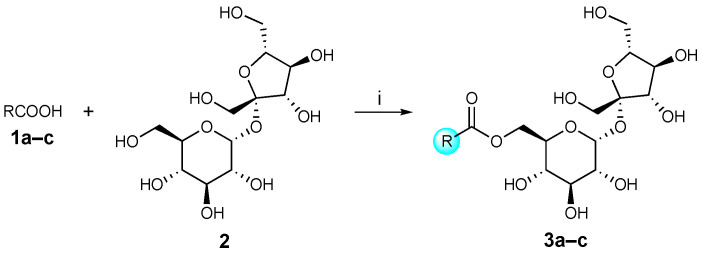
Reagents and conditions: (i) PPh_3_, DIAD, 0 °C, dry DMF, rt, 24 h.

*β-D-fructofuranosyl 6-O-(2-Phenylethanoyl)-α-D-glucopyranoside* (**3a**, *sucrose phenyl acetate, URB1480*) [38]

White solid. Yield: 50%. MS (ESI): 459 [M − H]^−^, 478 [M + NH_4_]^+^, 483 [M + Na]^+^. ^1^H NMR (DMSO-*d*_6_): δ = 3.07 (ddd, 1H, *J*_H4-OH4_ = 6.0 Hz, *J*_H4-H3_ ≈ *J*_H4-H5_ = 9.5 Hz, H^4^), 3.21 (ddd, 1H, *J*_H2-H1_ = 3.5 Hz, *J*_H2-OH2_ = 6.0 Hz, *J*_H2-H3_ = 9.5 Hz, H^2^), 3.39–3.43 (m, 2H, H^1′a^, H^1′b^), 3.50 (ddd, 1H, *J*_H3-OH3_ = 5.0 Hz, *J*_H3-H2_ ≈ *J*_H3-H4_ = 9.5 Hz, H^3^), 3.58–3.64 (m, 3H, H^5′^, H^6′a^, H^6′b^), 3.67 (d, *J* = 16.0 Hz, 1H, HC*H*Ar), 3.72 (d, *J* = 16.0 Hz, 1H, HC*H*Ar), 3.76–3.82 (m, 1H, H^4′^), 3.90 (dd, 1H, *J*_H3′-OH3′_ ≈ *J*_H3′-H4′_ = 8.0 Hz, H^3′^), 3.95 (m, 1H, H^5^), 4.05 (dd, 1H, *J*_H6a-H5_ = 6.0 Hz, *J*_H6a-H6b_ = 11.5 Hz, H^6a^), 4.28 (dd, 1H, *J*_H6b-H5_ = 1.5 Hz, *J*_H6b-H6a_ = 11.5 Hz, H^6b^), 4.43 (dd, 1H, *J*_OH6′-H6′a_ ≈ *J*_OH6′-H6′b_ = 5.5 Hz, OH^6′^), 4.60 (d, 1H, *J*_OH3′-H3′_ = 8.0 Hz, OH^3′^), 4.83 (dd, 1H, *J*_OH1′-H1′a_ ≈ *J*_OH1′-H1′b_ = 6.5 Hz, OH^1′^), 4.90 (d, 1H, *J*_OH3-H3_ = 5.0 Hz, OH^3^), 5.03 (d, 1H, *J*_OH4-H4_ = 6.0 Hz, OH^4^), 5.13 (d, 1H, *J*_OH2-H2_ = 6.0 Hz, OH^2^), 5.19 (d, 1H, *J*_H1-H2_ = 3.5 Hz, H^1^), 5.20 (d, 1H, *J*_OH4′-H4′_ = 6.0 Hz, OH^4′^), 7.24–7.35 (m, 5H, ArH) ppm. ^13^C NMR (DMSO-*d*_6_): δ = 21.2, 62.7, 63.1, 64.6, 70.6, 72.0, 73.1, 75.0, 77.4, 83.2, 92.0, 104.4, 127.2, 128.7, 129.9, 134.8, 171.7 ppm.

*β-D-fructofuranosyl 6-O-[2-(4-Phenyl)benzoyl]-α-D-glucopyranoside* (**3b**, *sucrose p-phenyl benzoate, URB1481*)

Pale yellow solid. Yield: 34%. MS (ESI): 521 [M − H]^−^, 540 [M + NH_4_]^+^, 545 [M + Na]^+^. ^1^H NMR (DMSO-*d*_6_) δ: 3.25–3.32 (m, 2H, H^4^, H^2^), 3.40–3.44 (m, 2H, H^1′a^, H^1′b^), 3.48 (ddd, 1H, *J*_H3-OH3_ = 5.0 Hz, *J*_H3-H2_ ≈ *J*_H3-H4_ = 9.0 Hz, H^3^), 3.52–3.62 (m, 3H, H^5′^, H^6′a^, H^6′b^), 3.78–3.83 (m, 1H, H^4′^), 3.92 (dd, 1H, *J*_H3′-OH3′_ ≈ *J*_H3′-H4′_ = 8.0 Hz, H^3′^), 4.09 (ddd, 1H, *J*_H5-H6b_ = 1.5 Hz, *J*_H5-H6a_ = 5.0 Hz, *J*_H5-H4_ = 9.0 Hz, H^5^), 4.36 (dd, 1H, *J*_H6a-H5_ = 5.0 Hz, *J*_H6a-H6b_ = 12.0 Hz, H^6a^), 4.41 (dd, 1H, *J*_OH6′-H6′a_ ≈ *J*_OH6′-H6′b_ = 6.0 Hz, OH^6′^), 4.47 (dd, 1H, *J*_H6b-H5_ = 1.5 Hz, *J*_H6b-H6a_ = 12.0 Hz, H^6b^), 4.68 (d, 1H, *J*_OH3′-H3′_ = 8.0 Hz, OH^3′^), 4.85 (dd, 1H, *J*_OH1′-H1′a_ ≈ *J*_OH1′-H1′b_ = 6.5 Hz, OH^1′^), 4.96 (d, 1H, *J*_OH3-H3_ = 5.0 Hz, OH^3^), 5.17 (d, 1H, *J*_OH4-H4_ = 5.0 Hz, OH^4^), 5.18 (d,1H, *J*_OH2-H2_ = 6.0 Hz, OH^2^), 5.21 (d, 1H, *J*_OH4′-H4′_ = 5.5 Hz, OH^4′^), 5.24 (d, 1H, *J*_H1-H2_ = 3.5 Hz, H^1^), 7.42–7.46 (m, 1H, ArH), 7.50–7.54 (m, 2H, ArH), 7.74–7.76 (m, 2H, ArH), 7.82–7.85 (m, 2H, ArH), 8.05–8.08 (m, 2H, ArH) ppm. ^13^C NMR (DMSO-*d*_6_) δ: 62.5, 63.0, 64.8, 70.5, 70.6, 72.0, 73.1, 74.9, 77.3, 83.1, 92.2, 104.5, 127.4, 127.5, 128.9, 129.0, 129.6, 130.4, 139.4, 145.1, 166.1 ppm.

*β-D-fructofuranosyl 6-O-[2-(4-Phenyl)phenylethanoyl]-α-D-glucopyranoside*, (**3c**, *sucrose p-biphenyl acetate, URB1482*)

White solid. Yield: 58%. ^1^H NMR (DMSO-*d*_6_) δ: 3.03 (ddd, 1H, *J*_H4-OH4_ = 6.0 Hz, *J*_H4_-_H3_ ≈ *J*_H4-H5_ = 9.5 Hz, H^4^), 3.13 (ddd, 1H, *J*_H2-H1_ = 3.5 Hz, *J*_H2-OH2_ = 6.0 Hz, *J*_H2-H3_ = 9.5 Hz, H^2^), 3.37–3.41 (m, 2H, H^1′a^, H^1′b^), 3.48 (ddd, 1H, *J*_H3-OH3_ = 5.0 Hz, *J*_H3-H2_ ≈ *J*_H3-H4_ = 9.5 Hz, H^3^), 3.56–3.63 (m, 3H, H^5′^, H^6′a^, H^6′b^), 3.76–3.81 (m, 1H, H^4′^), 3.88–3.96 (m, 2H, H^3′^, H^5^), 4.14 (dd, 1H, *J*_H6a-H5_ = 5.0 Hz, *J*_H6a-H6b_ = 11.5 Hz, H^6a^), 4.33 (dd, 1H, *J*_H6b-H5_ = 1.0 Hz, *J*_H6b-H6a_ = 11.5 Hz, H^6b^), 4.42 (dd, 1H, *J*_OH6′-H6′a_ ≈ *J*_OH6′-H6′b_ = 5.5 Hz, OH^6′^), 4.62 (d, 1H, *J*_OH3′-H3′_ = 8.0 Hz, OH^3′^), 4.82 (dd, 1H, *J*_OH1′-H1′a_ ≈ *J*_OH1′-H1′b_ = 6.0 Hz, OH1′), 4.89 (d, 1H, *J*_OH3-H3_ = 5.0 Hz, OH^3^), 5.02 (d, 1H, *J*_OH4-H4_ = 6.0 Hz, OH^4^), 5.13 (d, 1H, *J*_OH2-H2_ = 6.0 Hz, OH^2^), 5.17 (d, 1H, *J*_H1-H2_ = 3.5 Hz, H^1^) 5.20 (d, 1H, *J*_OH4′-H4′_ = 5.5 Hz, OH^4′^), 7.24–7.28 (m, 2H, ArH), 7.29–7.37 (m, 7H, ArH) ppm. ^13^C NMR (DMSO-*d*_6_): δ = 56.1, 62.5, 63.0, 64.6, 70.3, 70.4, 71.9, 73.1, 75.0, 77.4, 83.2, 92.0, 104.5, 127.4, 127.5, 128.9, 130.0, 139.48, 139.52, 172.4 ppm.

#### 3.2.2. General Procedures for the Synthesis of Lactose Tetra Acetal Aryl Aromatic and Aromatic Esters (**6a**–**c**), and Lactose Aryl Aromatic and Aromatic Ester Surfactants (**7a**–**c**, URB1419–1421) (Scheme 2)

Synthetic procedure, yields, MS, and ^1^H NMR and ^13^C NMR data were previously reported [21].

**Scheme 2:**
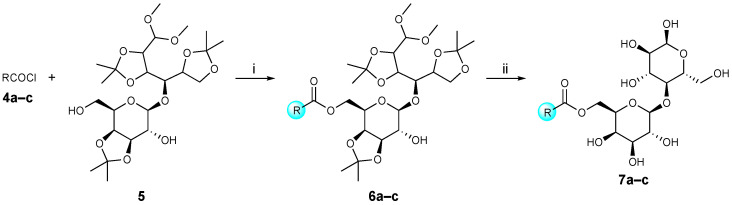
Reagents and conditions: (i) DIPEA, dry CH_2_Cl_2_, 0 °C, 1 h, then rt, 16 h; (ii) HBF_4_^.^Et_2_O, dry CH_3_CN, 30 °C, 3 h.

### 3.3. TGA and DTA/DSC Analyses

TGA and DTA/DSC of the surfactants were carried out using a simultaneous thermal analyzer STA 6000 (Perkin Elmer Inc., Waltham, MA, USA) at a heating rate of 10 °C/min from 35 °C to 650 °C in a nitrogen environment.

### 3.4. Fluorimetric Analysis

The pyrene emissions in the surfactant solutions at different concentrations (0.03–4 mM) were measured using a LS 55 fluorescence spectrometer (PerkinElmer, Waltar, MA, USA) equipped with a thermostatic bath HAAKE C25P (Artisan Scientific Corporation, Champaign, IL, USA). For the analyses, three microliters of the pyrene solution in CH_3_OH (2 μM) were added to each of the aqueous surfactant solutions. The excitation wavelength was 334 nm. Ten acquisitions were recorded for each solution in the range of 200–700 nm at a temperature of 25 °C. The peak intensity I (λ = 372 nm) to III (λ = 384 nm) ratio was plotted against the surfactant concentrations. CMC values were calculated from the center of the sigmoid by fitting the experimental data through the Boltzmann nonlinear regression (GraphPad Prism 6 software) according to the following equation:

$$\frac{bottom+\left(top-bottom\right)}{1+10^{\left[(\log CMC-x)hill\;slope\right]}}$$

where the top and bottom are the plateau of the curve and the hill slope is the steepness. 

### 3.5. DLS Measurement of the CMC

The scattering intensity to the detector (counts, kCps) was measured for different concentrations (0.03–4 mM) of surfactant solution using a Malvern Zetasizer Nano S (Malvern, Worcestershire, UK). Measurements were performed at 25 °C at a fixed laser position (4.65) and attenuation (11). CMC values were calculated from counts versus concentration plots by fitting the experimental data using the segmental linear regression model (GraphPad Prism 6 software).

### 3.6. Cytotoxicity Study MTT Cell Viability and LDH Release Assays

Calu-3 cells were seeded on 96-well plates at ~50,000 cells per well and were incubated to attain at least 80% confluence before the experiment. Prior to the assay, the culture medium was removed and replaced with different concentrations of surfactants (0.03 to 4.5 mM for the fluorescence spectroscopy and DLS analysis) in Hank’s balanced salt solution (HBSS). Triton X-100 (1% *v*/*v* in HBSS) and HBSS were used as the positive and negative control, respectively. After 3 h of incubation, the plate was centrifuged at 400× *g* for 5 min. The supernatant was collected for the LDH assay and the cell viability was measured with the MTT assay according to the manufacturers’ instructions, with at least three repeats for each sample.

### 3.7. TEER Measurement

Calu-3 cells were seeded on filter inserts Transwell^®^ at ~200,000 cells per insert. The cells were cultured in Dulbecco Modified Eagle Medium (DMEM)-F12 to confluence for around three weeks using an air–liquid culture condition. The culture medium was changed every 48 h. Prior to sample application, the culture medium was replaced with HBSS. Baseline TEER was recorded following 30 min of equilibration in HBSS. Surfactants at a concentration of 4 mM in HBSS were applied to the apical side of the cell monolayers, and HBSS was applied to the basolateral side. The cells were incubated with the samples or HBSS as a control for 3 h. During this period, TEER was measured every 30 min. The samples were then removed and the cells were washed extensively using HBSS. Culture medium was added to the basolateral chamber and a further measurement of TEER was taken at 24 h following sample application to establish TEER reversibility. The change in TEER was reported as a percentage relative to the baseline value.

### 3.8. In Vitro Cell Permeability Studies

Cell monolayers with TEER over 800 Ω were used in these studies to ensure the integrity of the monolayers. FITC-dextran MW 4000 was used as a model macromolecular drug. Prior to sample application, the culture medium was removed, and the cell layers were washed with HBSS. The cells were equilibrated in HBSS for 30 min. Sucrose ester solutions at 4 mM and dextran at a final concentration of 0.5 mg/mL in HBSS were applied to the apical side of the cells. HBSS was added to the basolateral side. A basolateral solution of 100 µL was collected at 30, 60, 90, 120, 150, and 180 min after sample application and replaced with an equivalent volume of fresh HBSS. The amount of dextran permeating the cell monolayers in 3 h was quantified by spectrofluorometric analysis. Dextran permeability is expressed as the apparent permeability coefficient, calculated using the following equation:

$$P_{app}=\left(\frac{\Delta Q}{\Delta t}\right)\times \left(\frac{1}{A\times C_{0}}\right)$$

where *P_app_* is the apparent permeability (cm/s), DQ/Dt is the permeability rate (amount of dextran traversing the cell layers over time), *A* is the diffusion area of the layer (cm^2^), and *C*_0_ is the apically added dextran concentration [39].

### 3.9. Statistics

All the data presented are the mean ± standard deviation of triplicate measurements and are representative of at least three independent experiments. The two-tailed paired Student’s *t*-test was used for the TEER and permeability studies. The results were considered significant at the level of *p* < 0.05.

## 4. Conclusions

The increasing need for permeation enhancers to deliver therapeutics across biological membranes (e.g., mucosal membranes) is a concern that needs to be addressed in the pharmaceutical and cosmetic fields. This is due to the fact that the difficulty in the permeation across biological membrane is a limitative step that decreases the bioavailability of the active pharmaceutical ingredients. In this work, we explored newly synthesized sucrose-based surfactants with potential application as permeation enhancers, comparing their results with those obtained by linear homologue lactose-based surfactants. The presented molecules show negligible cytotoxicity in vitro on Calu-3 cells at concentrations causing a moderate decrease in TEER (30–40% with the respect to the control) and a slight increase in the P_app_ coefficient. Moreover, in terms of permeability enhancement, URB1481 is the most effective as it induces a three-fold increase in FITC-dextran permeability across Calu-3 cells, despite the fact that its data are non-statistically significant when compared to the control. 

Overall, this study provides new insights into the potential pharmaceutical and cosmetic use of aromatic sugar-based surfactants as potential permeation enhancers endowed with a promising cytotoxicity profile. These results are supportive for the development of a new series of sugar-based surfactants as permeation enhancers for applications in different fields.

## Figures and Tables

**Table 1:** Calculated physicochemical properties of the sugar-based ester surfactants.

	Sugar Ester		MW	HLB ^a^	clogP ^b^
**3a**	URB1480	Sucrose phenyl acetate	460.4	12.9	−2.05
**3b**	URB1481	Sucrose *p*-phenyl benzoate	522.5	11.4	−0.32
**3c**	URB1482	Sucrose *p*-biphenyl acetate	536.5	11.1	−0.37
**7a**	URB1419	Lactose phenyl acetate	460.4	12.9	−2.05
**7b**	URB1420	Lactose *p*-phenyl benzoate	522.5	11.4	−0.32
**7c**	URB1421	Lactose *p*-biphenyl acetate	536.5	11.1	−0.37

^a^ Calculated Hydrophilic–Lipophilic Balance by Griffin’s method for non-ionic surfactants [24] [HLB = 20 × (MW hydrophilic portion/MW)]. ^b^ Calculated octanol–water portion coefficient clogP (by OSIRIS Property Explorer) [25].

**Table 2:** CMC values for sucrose-based surfactants (URB1480–URB1482) and lactose-based surfactants (URB1419–URB1421) as calculated from fluorescence and dynamic light scattering (DLS) measurements.

	Sugar Ester	Fluorescence Spectroscopy CMC (mM)	Dynamic Light Scattering CMC (mM)
**3a**	URB1480	0.861 ± 0.076	0.791 ± 0.045
**3b**	URB1481	0.305 ± 0.091	0.329 ± 0.023
**3c**	URB1482	0.095 ± 0.015	0.181 ± 0.026
**7a**	URB1419	0.753 ± 0.084	0.729 ± 0.075
**7b**	URB1420	0.270 ± 0.015	0.344 ± 0.034
**7c**	URB1421	0.110 ± 0.017	0.259 ± 0.063

## Data Availability

Data sharing not applicable.
